# Ion Channel Contributions to Morphological Development: Insights From the Role of Kir2.1 in Bone Development

**DOI:** 10.3389/fnmol.2020.00099

**Published:** 2020-06-09

**Authors:** Yunus H. Ozekin, Trevor Isner, Emily A. Bates

**Affiliations:** Department of Pediatrics, University of Colorado Anschutz Medical Campus, Aurora, CO, United States

**Keywords:** ion channel, bioelectricity, teratogen, nicotine, skeletal development

## Abstract

The role of ion channels in neurons and muscles has been well characterized. However, recent work has demonstrated both the presence and necessity of ion channels in diverse cell types for morphological development. For example, mutations that disrupt ion channels give rise to abnormal structural development in species of flies, frogs, fish, mice, and humans. Furthermore, medications and recreational drugs that target ion channels are associated with higher incidence of birth defects in humans. In this review we establish the effects of several teratogens on development including epilepsy treatment drugs (topiramate, valproate, ethosuximide, phenobarbital, phenytoin, and carbamazepine), nicotine, heat, and cannabinoids. We then propose potential links between these teratogenic agents and ion channels with mechanistic insights from model organisms. Finally, we talk about the role of a particular ion channel, Kir2.1, in the formation and development of bone as an example of how ion channels can be used to uncover important processes in morphogenesis. Because ion channels are common targets of many currently used medications, understanding how ion channels impact morphological development will be important for prevention of birth defects. It is becoming increasingly clear that ion channels have functional roles outside of tissues that have been classically considered excitable.

## Introduction

The correct development of a complex multicellular organism from a single fertilized egg requires cells communicating precisely. Genetic screens have identified some of the components of this cell-cell communication machinery including ligands, receptors, kinases, and transcription factors that influence cell fate and morphological development, thereby defining canonical developmental signaling pathways. The roles of many of these essential signaling components are conserved from *Caenorhabditis. elegans* and *Drosophila* to humans. Whole genome sequencing of human patients with morphological abnormalities has revealed a previously ignored class of genes that influences morphological development: ion channels. Mutations in the Kir2.1 potassium channel are associated with cleft palate, micrognathia, wide-set eyes, low-set ears, and digit abnormalities as part of Andersen-Tawil Syndrome (Plaster et al., 2001; Yoon et al., 2006). Disruptions in another potassium channel called Task3 (KCNK9) are associated with scoliosis, cleft palate and other characteristic facial features (Barel et al., 2008). Individuals with CaV1.2 calcium channel mutations present with similar craniofacial and digit abnormalities causing Timothy syndrome (Splawski et al., 2004; Diep and Seaver, 2015). UNC80 variants cause facial dysmorphisms and small hands and feet (Stray-Pedersen et al., 2016). Mutations in NALCN sodium channel are associated with facial dysmorphisms (Al-Sayed et al., 2013). TRPV4 calcium channel disruptions are associated with a wide variety of skeletal dysplasias (Nilius and Voets, 2013). Heterozygous or homozygous deletion of CHRNA7, a nicotinic acetyl choline receptor, is associated with facial dysmorphisms (Hoppman et al., 2013). Animal models have confirmed that loss of ion channel function influences development (Zaritsky et al., 2000; Dahal et al., 2012, 2017b; Ramachandran et al., 2013; George et al., 2019). Proper ion channel function is necessary in these morphological processes indiscriminate of ion channel class. Sodium, calcium, potassium, and chloride channels all play a role in development of various structures. This argues for a larger bioelectric mechanism that requires careful control of cellular membrane potential to correctly pattern a particular structure.

While human genetic syndromes that disrupt ion channel function and morphological development are rare, ion channels are common therapeutic targets in frequently prescribed medications. Many known teratogens, or agents that cause morphological changes in development, are known to affect ion channel function. For example, some of the most commonly used recreational drugs such as nicotine, marijuana, and alcohol bind and affect the function of ion channels. Heat is another known teratogen, and its effect on development could be mediated by ion channels. Here we review a selection of medications that target ion channels and affect development and discuss an example of how one particular ion channel influences bone development.

## Anti-Epilepsy Medications That Target Ion Channels Impact Development

Intrauterine exposure to some anti-epilepsy drugs (AEDs) that function as ion channel inhibitors is associated with increased incidence of congenital malformations. Prenatal exposures to topiramate, valproate, ethosuximide, phenobarbital, phenytoin, and carbamazepine are associated with significantly increased incidence of congenital malformations [reviewed in Veroniki et al. (2017)]. For example, exposure to phenytoin (Dilantin) during pregnancy can cause developmental abnormalities including growth deficiency, cleft lip and palate, congenital heart defects, abnormal finger and toe nails, genitourinary abnormalities, and neurological impairment that includes significant developmental delays. Similarly, intrauterine exposure to topiramate is associated with increased incidence of congenital defects such as cleft lip and palate (Blotiere et al., 2019). These medications have some overlapping targets, but all of them impact electrical activity of cells. Ethosuximide is a low voltage T-type calcium channel blocker (Coulter et al., 1989a, b). Carbamazepine and Phenytoin inhibit voltage-gated sodium channels (Meldrum and Rogawski, 2007). Phenobarbital inhibits GABA_A_ receptors (Sills and Brodie, 2001; Meldrum and Rogawski, 2007). Topiramate inhibits several types of channels including voltage-gated sodium channels, high voltage gated calcium channels, GABA receptors, and glutamate receptors. Shared developmental consequences of medications that have one shared activity and a second unshared activity suggest that it is the shared activity that is responsible for the developmental consequences.

The dose dependent correlation between developmental abnormalities and *in utero* exposure to Valproate (VPA) leading to a condition called Fetal Valproate Syndrome is well-documented. Valproate exposure is associated with a higher incidence in neural tube defects such as spina bifida, congenital heart defects, craniofacial abnormalities, limb defects, endocrine abnormalities, and genitourinary defects (Alsdorf and Wyszynski, 2005; Artama et al., 2005; Schorry et al., 2005). Craniofacial defects are more prevalent in babies exposed to VPA during the first trimester and include thin or cleft lip, cleft palate, tall forehead, flat nasal bridge, broad nasal root, shallow philtrum, medial eyebrow deficiency, and microcephaly (Clayton-Smith and Donnai, 1995). In addition, exposure to VPA during gestation results in higher incidence of autism spectrum disorder, cognitive impairment, and developmental delay (Kozma, 2001; Roullet et al., 2013). In utero exposure of mice to valproate provides evidence that the developmental effects of valproate are causative. For example, a 1-day exposure to VPA during gestation caused structural heart abnormalities (Wu et al., 2010; Philbrook et al., 2019). The mechanism that underlies how valproate affects development has not been well characterized. Valproate blocks voltage-gated sodium channels and increases gamma-aminobutyric acid- (GABA) mediated neurotransmission (Rosenberg, 2007). Additionally, Table 1 represents valproate exposure affects the Wnt and ERK pathways which would adversely affect development, but the direct consequences of valproate affecting these pathways are unknown (Rosenberg, 2007). Valproate and topiramate also inhibit histone deacetylases (HDACs) and some have proposed that this is the mechanism by which developmental pathways are affected and birth defects arise (Phiel et al., 2001; Gurvich et al., 2005). However, ethosuximide, phenytoin, phenobarbital, and carbamazepine do not inhibit HDACs, but similarly disrupt morphological development (Eyal et al., 2004; Veroniki et al., 2017). Because ethosuximide, phenytoin, phenobarbital, carbamazepine, and valproate all impact a cell’s potential for electrical activity, we suggest that changing a cell’s membrane potential may be a cause of their teratogenic influence on development.

**TABLE 1:** Congenital birth defects found in association with intrauterine exposure to anti-epileptic medications that inhibit ion channels.

Anti-epileptic medication	Ion channels inhibited	Associated defects
Phenobarbital	GABA_A_ receptors	Cleft lip, cleft palate, reduced head circumference, congenital heart defects, and reduced birth weight
Phenytoin	Voltage-gated Calcium channels	Cleft lip, cleft palate, reduced head circumference, wide mouth, low hair line, congenital heart defects, reduced birth weight, abnormal finger, and toe nails, genitourinary abnormalities
Ethosuximide	Low voltage-gated T type calcium channels	Cleft palate, cleft lip, and limb abnormalities (club foot)
Valproate	Voltage gated calcium channels and HDACs	Cleft lip, cleft palate, flat nasal bridge, broad nasal root, shallow philtrum reduced head circumference, cardiac abnormalities, Neural tube defects, reduced birth weight, limb defects, and genitourinary abnormalities (hypospadia in males)
Topiramate	Voltage gated sodium channels, high voltage gated calcium channels, GABA receptors, glutamate receptors, and HDACs	Cleft lip, cleft palate, and hypospadia

## Heat as a Developmental Teratogen

Heat is detected by heat-sensitive ion channels that include TRPV family members TRPV1 (>42°C), TRPV2 (>52°C), TRPV3 (34–38°C), and TRPV4 (27–35°C), Heat was first implicated as a teratogen when a wave of birth defects and abortions in a colony of guinea pigs that had been accidentally exposed to high temperatures was noticed (Edwards, 1967). These effects were subsequently observed in other systems including mouse, rat, chick, sheep, and non-human primates [reviewed in Edwards et al. (2003)]. The temperature-sensitivity ranges vary from animal to animal as some organisms have adapted to function at higher or lower temperatures. An analysis of data from the National Birth Defect Prevention study associated maternal cold or flu with accompanied fever to various birth defects (anencephaly, spina bifida, encephalocele, cleft lip with or without cleft palate, colonic atresia/stenosis, bilateral renal agenesis/hypoplasia, limb reduction defects, and gastroschisis) while maternal cold or flu without fever was not associated with the listed defects (Waller et al., 2018). Although the impacts of maternal hyperthermia on embryonic development have been well-documented, the mechanisms are less understood. Several studies exist linking the effects of maternal hyperthermia to disruptions in cellular processes like heat shock response (Bennett et al., 1990), migration (Upfold et al., 1991) cell survival (Shiota, 1988) and gene expression (Hosako et al., 2009). The upstream control of these processes has not been determined. Recently, a study demonstrated that maternal fever associated craniofacial and heart defects could be modulated through control of two heat-activated transient receptor potential (TRP) ion channels in the neural crest cells of chick embryos (Hutson et al., 2017). Pharmacological inhibition of TRPV1 shielded the embryo from observed defects under heat exposure. Conversely, activation of TRPV1 or TRPV4 with chemical agonists alone was sufficient to induce craniofacial and cardiac phenotypes without heat. The upstream activation of ion channels may serve as the regulatory point to which other observed cellular effects are controlled, but further research is needed to understand how these channels are specifically affecting neural crest-derived tissues. Additionally, these same mechanisms may exist as the mode of pathogenesis of other structural defects (i.e., neural tube defects).

Cold is detected by cold-sensitive channels including TRPM8 (25–28°C) and TRPA1 (<17°C) (Belmonte and Viana, 2008). Although maternal fever and hyperthermia is more common, hypothermia can also cause birth defects. Exposing chick embryos to sustained periods of hot or cold results in congenital malformations such as microphthalmia, gastroschisis, hyperlordosis, or exencephaly (Peterka et al., 1996). It can be hypothesized that birth defects may be due to upstream activation and control of these channels, but this argument requires further investigation.

## Nicotine as a Developmental Teratogen

Nicotine binds and affects the activity of several ion channels and therefore influences excitability or membrane potential of cells that express those ion channels. Nicotine has long been acknowledged as a neuroteratogen (Levin and Abreu-Villaça, 2018). However, its effects expand far beyond that of the central nervous system including the developing cardiovascular (Lawrence et al., 2008) respiratory (Gibbs et al., 2016) endocrine (Tweed et al., 2012) and reproductive (Budin et al., 2017) systems. Along with these effects, nicotine is also associated with increased incidence of other congenital birth defects. A systemic review of maternal smoking and birth defects revealed significant positive associations with a range of malformations including cardiovascular defects, musculoskeletal defects, limb/digit defects, clubfoot, craniosynostosis, facial defects, orofacial clefts, and others (Hackshaw et al., 2011). Many studies of developmental toxicity of nicotine are conducted through the lens of maternal smoking. This can confound results because there are numerous known cytotoxic components of cigarettes outside of nicotine (Talhout et al., 2011). Yet human studies on smokeless tobacco also show detrimental effects, implicating nicotine as a causative agent in many of these processes (Wikstrom et al., 2010; Baba et al., 2012) as opposed to the other chemicals taken in from smoking cigarettes. Both the cardiovascular and musculoskeletal systems are reliant on ion channels for proper function and development (Barchi, 1997; Rahm et al., 2018). Recent work shows that nicotine alone can increase proliferation of murine calvarial cells providing a possible mechanism for the increased observation of craniosynostosis in nicotine-exposed fetuses (Durham et al., 2019). They also show that several nicotinic acetylcholine receptor subunits (α3, α7, β2, β4) are present in the calvarial sutures and synchondroses. Nicotine exposure additionally affects other bone structures, such as the mandible, in which a reduction in mandibular ramus height, mandibular body height, and molar length can be observed (Durham et al., 2019). Additionally, e-cigarette aerosol exposure in *Xenopus* has recently been shown to cause craniofacial defects including midface hypoplasia and median facial clefts. Although the craniofacial defects observed in *Xenopus* can be seen through exposure with e-cigarette aerosol alone, addition of nicotine exacerbates these effects (Kennedy et al., 2017).

Aside from nicotine’s capacity to bind as an agonist to nicotinic acetylcholine receptors, it is also able to interact with other ion channels. Human cleft lip and palate fibroblasts and normal human fibroblasts exposed to nicotine show transcriptional intersections of several molecular signaling pathways including those of TGF-β, retinoic acid, and GABA-ergic signaling (Baroni et al., 2010). Nicotine can directly bind and completely block inwardly rectifying potassium (Kir) channel activity (Wang et al., 2000). Kir channel subunits, such as Kir2.1, have been implicated in craniofacial and skeletal development, and particularly palatogenesis, through disruptions in BMP signaling, which is part of the TGF-β superfamily (Belus et al., 2018). Nicotine partially blocks activity of hyperpolarization-activated cyclic nucleotide–gated (HCN) channels (Griguoli et al., 2010). The neuroteratogenic effects of nicotine in *Xenopus* can be reversed through exogenous HCN2 expression (Pai et al., 2018). Because HCN2 is only partially blocked by nicotine, exogenous expression of HCN2 returns the endogenous membrane potential to levels similar to that of an unexposed embryo. Remarkably, not only are structural defects repaired, but also cognitive learning ability. These data support the argument that disruption of development can occur by perturbing the endogenous bioelectric patterning of a tissue. Under this principle, it is less important as to *which* channels are being distrupted and more important as to *how* those disruptions affect the prepatterned bioelectric state of that tissue. This holds promise to using therapeutics as substances which could act in opposite directions (i.e., depolarizing and repolarizing) and could be used in conjunction to correct developmental deficits.

## Cannabinoids as a Developmental Teratogen

As the use of marijuana, tetrahydrocannabinol (THC), and cannabidiol (CBD) products become more popular and several states have proceeded with their legalization, it would be amiss to not consider their potential as morphological teratogens. The state of Colorado in the United States of America, has shown an increase in incidence of congenital birth defects and anomalies since the period of cannabis legalization (Reece and Hulse, 2019). A sizable body of work has been published looking at cannabis and cannabinoids in the context of neurodevelopment (Persaud and Ellington, 1967; Psychoyos et al., 2012; De Salas-Quiroga et al., 2015; Richardson et al., 2016). Emerging work has also implicated these substances in morphogenesis. It was recently shown that several cannabinoids can cause developmental defects such as coloboma, exencephaly, philtrum deficiency, and cleft anterior palate in both mice and zebrafish (Fish et al., 2019). Interestingly, these phenotypes mimic those of fetal alcohol syndrome disorder. The authors go on to demonstrate that combined exposure of cannabinoids coupled with alcohol exposure increases the incidence of these defects and works through a CB1-Hedgehog interaction. Furthermore, it has been reported that CBD can bind TRP channels which, as discussed earlier, can cause craniofacial defects (Hutson et al., 2017; Muller et al., 2018). TRPV4 mutations also cause skeletal dysplasias through aberrant calcium signaling. This produces BMP-inhibiting Follistatins that prevent chondrocytes from undergoing hypertrophy to form bone (Leddy et al., 2014a, b). Transient activation of TRPV4 channels in utero through cannabinoids could produce similar effects depending on timing and dosage. In fact, cannabinoids bind a range of other ion channels outside of their specific CB1 and CB2 receptors including voltage-gated sodium (Na_v_), voltage-gated potassium (K_v_), and ATP-gated potassium channels (K_ATP_) (Watkins, 2019). Though we do not yet understand to what extent all these channels specifically intersect in the context of morphogenesis, disrupting the endogenous bioelectric landscape of a tissue is predicted to have negative outcomes (Pai et al., 2018). Therefore, binding of these substances to ion channels warrants further investigation into their developmental consequences.

## Role of Ion Channels in Bone and Cartilage Development

Teratogens that can bind and affect ion channel function alter bone and cartilage development in the head and limbs. This results in birth defects like cleft palate, craniosynostosis, and digit abnormalities. To better understand how information gained from ion channels can be applied toward comprehending complex problems in morphogenesis, we discuss the potassium channel Kir2.1 and its role in skeletal development. A brief overview of bone formation is necessary before discussing the role of ion channels in such a process.

The formation of bone tissue can be categorized into endochondral ossification and intramembranous ossification. Intramembranous ossification is responsible for the formation of flat bones, while endochondral ossification forms the weight bearing bones long bones. Intramembranous ossification occurs through the condensation of neural crest derived mesenchymal cells. These cells develop into osteoblasts that secrete a collagen-proteoglycan (osteoid) matrix that will give rise to the mature bone cells, or osteocytes. Bone morphogenetic proteins (BMP) induce osteocyte fate and promote bone formation. For endochondral ossification, developed cartilage tissue is replaced by bone tissue. Mesenchymal cells differentiate into chondrocytes that secrete a cartilage matrix to form a scaffold that is replaced by mature bone tissue. Chondrocytes then undergo hypertrophy to allow the mineralization of the matrix. The surrounding cells begin their transition to osteoblasts which replace the hyaline cartilage with bone tissue (Bruder and Caplan, 1989).

Ion channels regulate resting membrane potential for correct bone and cartilage development, [reviewed in An (2019)]. TRPV4 regulates cartilaginous osmotic fluctuations (An, 2019). The voltage-gated calcium channel CaV1.2 promotes bone formation (Cao et al., 2017, 2019). A human Timothy Syndrome variant of CaV1.2 expressed in an ovariectomy-induced osteoporosis model prevents estrogen deficiency induced bone loss (Cao et al., 2017). Piezo1, a mechanosensitive ion channel, is necessary for bone formation (Sun et al., 2019). The inwardly rectifying potassium channel Kir2.1 impacts BMP signaling, which is important for osteoblasts and osteoclast differentiation (Dahal et al., 2012; Pai et al., 2018; Nguyen et al., 2013). We have known that voltage, calcium, and stretch activated ion channels are expressed in bone cells for decades, but their functions in bone development are less clear (Ypey et al., 1992). Insights from one ion channel, Kir2.1, may provide some clues.

### Kir2.1 and Its Role in Bone Development

Mutations that disrupt the inwardly rectifying potassium channel (Kir2.1), encoded by the *KCNJ2* gene, cause Andersen-Tawil syndrome. Patients with this syndrome have higher incidence of digital abnormalities, as well as cleft palate and other craniofacial abnormalities (Nguyen et al., 2013; Simkin et al., 2018). Cell culture work using Andersen Syndrome induced pluripotent stem cell mesenchymal stem cells (AS-IPSC-MSCs) revealed lower chondrogenic differentiation potential compared to wild type cells (Sacco et al., 2015; Pini et al., 2018). *KCNJ2* knock out (KO) mice have severe craniofacial defects (Dahal et al., 2012, 2017a; Belus et al., 2018; George et al., 2019). In mice, the *KCNJ2* gene is expressed in the fusing midline and anterior neural tissues of E8.5 embryos. This expression continues through E9.5, when expression can clearly be seen in several craniofacial structures, including the frontonasal prominence and the first and second branchial arches (Adams et al., 2016). Loss of Kir2.1 function also impacts craniofacial development in Xenopus and Kir2.1 is present in the anterior neural folds of stage 14 and 17 at stage 27. Together, these data suggest that Kir2.1 is necessary for proper craniofacial development and has a conserved role among vertebrates including humans, mice, and *Xenopus.*

Kir2.1 is important for bone morphogenetic protein (BMP) signaling as genetic disruptions in the channel lead to decreased activation of downstream BMP targets. BMP is secreted from a cell, which can bind to a complex of type 1 and type 2 serine-threonine kinase receptors. The type 2 receptor phosphorylates the type 1 receptor which in turn phosphorylates SMAD proteins that complex with co-SMADs to enter the nucleus where they induce gene expression (Heldin et al., 1997; Belus et al., 2018; Lowery and Rosen, 2018). Although flies do not have skeletons, studying this process in *Drosophila* has provided insights into the mechanism of Kir2.1’s effect of BMP signaling (Dahal et al., 2012; Sacco et al., 2015; Belus et al., 2018; Pini et al., 2018). Ablation of a Kir2.1 homolog, Irk2, in *Drosophila* wing disks results in defects in several structures including bristles, veins, and wing which phenocopy a loss of a *Drosophila* BMP called Decapentaplegic (Dpp) (Dahal et al., 2012, 2017b). Inhibition of Irk2 reduces Dpp signaling and changes the dynamics of Dpp release in the developing wing from a pulsatile manner to a continuous one (Dahal et al., 2012, 2017a). Similarly in mice, genetic Kir2.1 ablation phenocopies BMP2/4 mutants, resulting in severe craniofacial phenotypes including enlarged fontanelle, hypoplastic mandible, hypoplastic nasal bones, and cleft palate as well as limb and digit defects (Suzuki et al., 2009; Bonilla-Claudio et al., 2012; Sacco et al., 2015; Belus et al., 2018; Pini et al., 2018; Chen et al., 2019). Phenotypic similarities in mice with genetic ablation of BMP2/4 or Kir2.1 suggest that BMPs and Kir2.1 are required for the same developmental processes (Dahal et al., 2017b; Belus et al., 2018). Upon homozygous deletion of Kir2.1 in developing mice, a reduction in BMP signaling is observed in the palate shelves of E13.5 embryos (Dahal et al., 2012, 2017a; Belus et al., 2018). Interestingly, there is not a difference in mRNA levels of TGF-beta superfamily ligands, receptors, inhibitors, or intracellular components of the pathway suggesting that this is due to a deficit in cell-cell communication and BMP release dynamics as opposed to production of signaling components.

One possible model to explain how ion channels such as Kir2.1 could disrupt BMP signaling is that release of BMP is regulated by membrane potential and intracellular calcium concentrations. In support of this model, inhibition of Irk2 alters intracellular calcium dynamics in developing wings of *Drosophila* (Dahal et al., 2012, 2017a). This is reminiscent of how ion channels regulate secretion of signaling molecules from neurons and pancreatic β-cells. Both of these examples rely on ion channel dependent calcium concentrations to drive fusion of vesicles to cellular membranes for secretion of molecules. Very little is known about how BMP release is controlled. Differences in cellular voltage could result in changes in timing or intensity of signals. If membrane potential regulates release of morphogens, we would expect that multiple ion channels would contribute to morphogenesis. Indeed, an ion channel knockout/RNAi screen in *Drosophila* revealed that ion channels across classes contribute to wing morphogenesis (George et al., 2019). Many of these channels have human orthologs and are associated with morphological defects in human patients. While we know that certain ion channels impact BMP signaling to affect morphological development, this model may potentially be extended to other secreted morphogens. Further research is needed to determine the details of these mechanisms and better understand how bioelectrical networks regulate morphogenesis.

## Conclusion

The discovery that ion channels actively participate in morphogenesis has opened up a new topic of developmental research: bioelectricity. Understanding how endogenous bioelectric patterns regulate developmental signaling, both at the cellular and tissue levels, has become a key question. Individuals with genetic mutations in ion channels can have dysmorphic facial features and limbs such as those patients with Andersen-Tawil, Birk-Barel, and Timothy syndrome. However, these syndromes are rare. Pharmaceuticals that target ion channels necessitate research that elucidates bioelectricity in development. Behind rhodopsin-like G protein-coupled receptors (GPCRs) and nuclear receptors, ligand and voltage-gated ion channels are the third and fourth most common drug targets (Overington et al., 2006). Understanding the impacts of these drugs on a developing embryo is crucial to reducing incidence of birth defects. Indeed, we have already seen how some ion-channel targeting drugs like the anti-epilepsy medication, valproate, can cause birth defects. Additionally, other substances that influence ion channels such as nicotine and cannabinoids can cause similar defects. Even environmental effects like heat and cold, when taken to extremes, can cause birth defects potentially through ion-channel mediated mechanisms. Extensive work looking at the Kir2.1 potassium channel shows that a single channel can disrupt coordinated biological processes. Bone formation, discussed in this review, can be greatly perturbed through alteration of the electrical potential of the developing tissue, thereby causing downstream signaling deficits. Understanding how these channels contribute to developmental processes, as well as how teratogens can interact with these ion channels to affect those processes is crucial to understanding morphogenesis. We are still far from understanding how ion channels, collectively interact to coordinate proper development.

## Author Contributions

All authors listed have made a substantial, direct and intellectual contribution to the work, and approved it for publication.

## Conflict of Interest

The authors declare that the research was conducted in the absence of any commercial or financial relationships that could be construed as a potential conflict of interest. The handling editor declared a past collaboration with one of the authors EB.
